# Supplementary material for: Modeling glioblastoma heterogeneity as a dynamic network of cell states
Source: Mol Syst Biol. 2021 Sep 16;17(9):e10105. doi: 10.15252/msb.202010105 (PMC8444284; doi:10.15252/msb.202010105)
Supplement: Supplementary file 6 — Source Data for Figure 5 [file MSB-17-e10105-s004.zip › Figure5A_sourcedata/GSEA_3017/nosigdb_stateC.GseaPreranked.1622795002785/GAROFANO_IAVARONE_PPR_SUBTYPE.html]

Details for gene set GAROFANO\_IAVARONE\_PPR\_SUBTYPE[GSEA]

|  || Dataset | state13017 |
| Phenotype | NoPhenotypeAvailable |
| Upregulated in class | na\_pos |
| GeneSet | GAROFANO\_IAVARONE\_PPR\_SUBTYPE |
| Enrichment Score (ES) | 0.70242935 |
| Normalized Enrichment Score (NES) | 5.7126584 |
| Nominal p-value | 0.0 |
| FDR q-value | 0.0 |
| FWER p-Value | 0.0 |
Table: GSEA Results Summary

  

Fig 1: Enrichment plot: GAROFANO\_IAVARONE\_PPR\_SUBTYPE      
 Profile of the Running ES Score & Positions of GeneSet Members on the Rank Ordered List

  

| PROBE | GENE SYMBOL | GENE\_TITLE | RANK IN GENE LIST | RANK METRIC SCORE | RUNNING ES | CORE ENRICHMENT || 1 | ATAD2 |  |  | 7 | 0.649 | 0.0096 | Yes |
| 2 | FAM111B |  |  | 8 | 0.616 | 0.0314 | Yes |
| 3 | HIST1H4C |  |  | 9 | 0.603 | 0.0528 | Yes |
| 4 | EZH2 |  |  | 14 | 0.574 | 0.0654 | Yes |
| 5 | CLSPN |  |  | 17 | 0.561 | 0.0815 | Yes |
| 6 | RRM2 |  |  | 19 | 0.542 | 0.0988 | Yes |
| 7 | HMGB2 |  |  | 20 | 0.534 | 0.1178 | Yes |
| 8 | XRCC2 |  |  | 26 | 0.495 | 0.1257 | Yes |
| 9 | CHAF1A |  |  | 27 | 0.494 | 0.1432 | Yes |
| 10 | UBE2T |  |  | 31 | 0.475 | 0.1543 | Yes |
| 11 | HIST1H1B |  |  | 35 | 0.460 | 0.1649 | Yes |
| 12 | GMNN |  |  | 38 | 0.453 | 0.1771 | Yes |
| 13 | ESCO2 |  |  | 43 | 0.440 | 0.1850 | Yes |
| 14 | RAD51AP1 |  |  | 46 | 0.435 | 0.1966 | Yes |
| 15 | PCNA |  |  | 48 | 0.435 | 0.2101 | Yes |
| 16 | TMPO |  |  | 49 | 0.432 | 0.2254 | Yes |
| 17 | DTL |  |  | 50 | 0.430 | 0.2407 | Yes |
| 18 | TOP2A |  |  | 52 | 0.426 | 0.2539 | Yes |
| 19 | SPC25 |  |  | 53 | 0.415 | 0.2686 | Yes |
| 20 | BRCA1 |  |  | 54 | 0.410 | 0.2831 | Yes |
| 21 | WDR76 |  |  | 56 | 0.403 | 0.2955 | Yes |
| 22 | TYMS |  |  | 60 | 0.390 | 0.3036 | Yes |
| 23 | RRM1 |  |  | 62 | 0.386 | 0.3154 | Yes |
| 24 | SMC2 |  |  | 63 | 0.386 | 0.3291 | Yes |
| 25 | NASP |  |  | 75 | 0.371 | 0.3211 | Yes |
| 26 | POLD3 |  |  | 76 | 0.367 | 0.3341 | Yes |
| 27 | CENPH |  |  | 77 | 0.366 | 0.3471 | Yes |
| 28 | SMC4 |  |  | 78 | 0.365 | 0.3601 | Yes |
| 29 | CDCA5 |  |  | 81 | 0.361 | 0.3690 | Yes |
| 30 | GINS2 |  |  | 83 | 0.360 | 0.3799 | Yes |
| 31 | KLHL23 |  |  | 84 | 0.359 | 0.3926 | Yes |
| 32 | STMN1 |  |  | 85 | 0.357 | 0.4053 | Yes |
| 33 | BARD1 |  |  | 86 | 0.357 | 0.4179 | Yes |
| 34 | DUT |  |  | 87 | 0.356 | 0.4306 | Yes |
| 35 | EPB41 |  |  | 88 | 0.355 | 0.4432 | Yes |
| 36 | MCM7 |  |  | 91 | 0.352 | 0.4518 | Yes |
| 37 | MAP2 |  |  | 96 | 0.345 | 0.4564 | Yes |
| 38 | ATAD5 |  |  | 97 | 0.345 | 0.4686 | Yes |
| 39 | FEN1 |  |  | 98 | 0.342 | 0.4807 | Yes |
| 40 | NUSAP1 |  |  | 100 | 0.340 | 0.4909 | Yes |
| 41 | NCAPD3 |  |  | 103 | 0.334 | 0.4988 | Yes |
| 42 | ZNF43 |  |  | 107 | 0.330 | 0.5048 | Yes |
| 43 | H2AFX |  |  | 115 | 0.322 | 0.5027 | Yes |
| 44 | HIRIP3 |  |  | 116 | 0.321 | 0.5141 | Yes |
| 45 | TIMELESS |  |  | 119 | 0.319 | 0.5216 | Yes |
| 46 | SMC1A |  |  | 125 | 0.312 | 0.5231 | Yes |
| 47 | HMGN2 |  |  | 126 | 0.312 | 0.5341 | Yes |
| 48 | DNMT1 |  |  | 127 | 0.311 | 0.5452 | Yes |
| 49 | DPF1 |  |  | 134 | 0.303 | 0.5444 | Yes |
| 50 | KIF15 |  |  | 136 | 0.302 | 0.5532 | Yes |
| 51 | NCAPG |  |  | 137 | 0.301 | 0.5638 | Yes |
| 52 | CENPK |  |  | 139 | 0.299 | 0.5725 | Yes |
| 53 | LMNB1 |  |  | 142 | 0.293 | 0.5790 | Yes |
| 54 | ASF1B |  |  | 143 | 0.291 | 0.5894 | Yes |
| 55 | HNRNPAB |  |  | 144 | 0.290 | 0.5996 | Yes |
| 56 | RFC3 |  |  | 156 | 0.283 | 0.5885 | Yes |
| 57 | CEP78 |  |  | 159 | 0.281 | 0.5947 | Yes |
| 58 | ZNF93 |  |  | 160 | 0.281 | 0.6047 | Yes |
| 59 | HIST2H2AC |  |  | 161 | 0.281 | 0.6146 | Yes |
| 60 | PARP1 |  |  | 163 | 0.280 | 0.6226 | Yes |
| 61 | MYBL2 |  |  | 164 | 0.278 | 0.6325 | Yes |
| 62 | HELLS |  |  | 171 | 0.271 | 0.6306 | Yes |
| 63 | MAD2L1 |  |  | 174 | 0.270 | 0.6363 | Yes |
| 64 | RIF1 |  |  | 178 | 0.266 | 0.6400 | Yes |
| 65 | POLA1 |  |  | 179 | 0.266 | 0.6494 | Yes |
| 66 | MSH2 |  |  | 180 | 0.265 | 0.6588 | Yes |
| 67 | DDX11 |  |  | 181 | 0.265 | 0.6682 | Yes |
| 68 | RMI1 |  |  | 183 | 0.263 | 0.6756 | Yes |
| 69 | NCAPG2 |  |  | 187 | 0.260 | 0.6790 | Yes |
| 70 | MCM4 |  |  | 189 | 0.259 | 0.6863 | Yes |
| 71 | CDK1 |  |  | 191 | 0.255 | 0.6934 | Yes |
| 72 | RSBN1L |  |  | 192 | 0.254 | 0.7024 | Yes |
| 73 | TNFAIP8L1 |  |  | 215 | -0.257 | 0.6693 | No |
| 74 | CREB5 |  |  | 309 | -0.281 | 0.5004 | No |
| 75 | SLIT1 |  |  | 446 | -0.349 | 0.2512 | No |
| 76 | NTRK3 |  |  | 464 | -0.368 | 0.2316 | No |
| 77 | PCDH10 |  |  | 565 | -0.573 | 0.0596 | No |
Table: GSEA details [plain text format]

  

Fig 2: GAROFANO\_IAVARONE\_PPR\_SUBTYPE: Random ES distribution      
 Gene set null distribution of ES for **GAROFANO\_IAVARONE\_PPR\_SUBTYPE**
